# KSRV: a Kernel PCA-Based framework for inferring spatial RNA velocity at single-cell resolution

**DOI:** 10.3389/fgene.2025.1695803

**Published:** 2025-11-07

**Authors:** Yan He, Jian Jiang, Huahai Qiu, Ya-Zhou Shi, Ben-Gong Zhang

**Affiliations:** 1 School of Mathematics and Statistics, Wuhan Textile University, Wuhan, China; 2 Center for Applied Mathematics and Interdisciplinary Sciences, Wuhan Textile University, Wuhan, China

**Keywords:** RNA velocity, scRNA-seq data, cell differentiation, Kernel PCA, data integration

## Abstract

Understanding the temporal dynamics of gene expression within spatial contexts is essential for deciphering cellular differentiation. RNA velocity, which estimates the future state of gene expression by distinguishing spliced from unspliced mRNA, offers a powerful tool for studying these dynamics. However, current spatial transcriptomics technologies face limitations in simultaneously capturing both spliced and unspliced transcripts at high resolution. To address this challenge, a novel computational framework called KSRV (Kernel PCA–based Spatial RNA Velocity) that integrates single-cell RNA-seq with spatial transcriptomics using Kernel Principal Component Analysis. It enables accurately inference of RNA velocity in spatially resolved tissue at single-cell resolution. KSRV was validated by using 10x Visium data and MERFISH datasets. The results demonstrate its both accuracy and robustness comparing with the existed method such as SIRV and spVelo. Furthermore, KSRV successfully revealed spatial differentiation trajectories in the mouse brain and during mouse organogenesis, highlighting its potential for advancing our understanding of spatially dynamic biological processes.

## 1 Introduction

Cell differentiation dynamics research is of great significance for understanding biological development, disease occurrence, and regenerative medicine (Saliba et al., 2014; Chen et al., 2019). However, traditional single-cell RNA sequencing (scRNA-seq) technology only provides static snapshots of gene expression in cells, failing to directly capture the dynamic changes in cell status and differentiation trajectories, and has limitations in determining cell fate directions (Bergen et al., 2020; Gao et al., 2022; Li et al., 2024). Trajectory inference algorithms aim to recon-struct cell development sequences and differentiation paths from static data by constructing potential branching trajectories based on transcriptome similarity (Haghverdi et al., 2016; Qiu et al., 2017; Street et al., 2018; Setty et al., 2019; Wolf et al., 2019; Zhou et al., 2024). In recent years, the introduction of RNA velocity theory (La Manno et al., 2018; Li et al., 2023) has brought a breakthrough to trajectory inference, enabling the inference of gene expression trends by analyzing the abundance of unspliced and spliced mRNA, thus providing a robust method for trajectory inference and offering dynamic information on the direction of cell differentiation trajectories and cell fate predictions.

Although RNA velocity analysis has been widely applied to various scRNA-seq datasets, most current methods are limited to isolated cells and neglect the spatial location of cells within tissues (Pham et al., 2023). However, spatial tissue architecture plays a crucial role in differentiation, as signaling pathways, gene expression patterns, and developmental trajectories can vary significantly across different microenvironments. Spatial transcriptomics has transformed our understanding of complex biological systems by enabling gene expression profiling with preserved spatial context. For instance, integrating RNA velocity with spatial information makes it possible to investigate the spatiotemporal dynamics of cell differentiation and improve the accuracy of cell fate prediction. (Burgess, 2019; Moses and Pachter, 2022; Wang et al., 2024).

Spatial transcriptomics techniques (Shah et al., 2016; Eng et al., 2019; Rodriques et al., 2019; Gyllborg et al., 2020; Stickels et al., 2020) provide rich tissue spatial expression profiles, but often lack spliced/unspliced transcripts, limiting their direct application to RNA velocity analysis. To address this, several approaches have attempted to align scRNA-seq data with spatial transcriptomics data to complement the spatial expression patterns (Stuart et al., 2019; Welch et al., 2019; Shengquan et al., 2021), providing possibilities for spatial RNA velocity inference. These integration methods generally fall into two categories: deconvolution and mapping (Yan et al., 2024). Deconvolution methods aim to estimate the cell-type composition or average gene expression at each spatial location but often ignore cell-level resolution (Elosua-Bayes et al., 2021; Cable et al., 2021; Kleshchevnikov et al., 2022; Li et al., 2022). Mapping-based methods, such as SpaGE (Abdelaal et al., 2020), typically perform dimensionality reduction separately on scRNA-seq and spatial transcriptomics data, and then project spatial spots into the low-dimensional embedding learned from scRNA-seq. The gene expression of each spot is then inferred by aggregating information from its nearest single-cell neighbors in the latent space. While effective for predicting missing genes, these approaches often rely on linear dimensionality reduction techniques such as PCA, which may not capture complex nonlinear relationships between modalities. Moreover, they are primarily designed for gene imputation and rarely address the inference of spatial RNA velocity.

In this study, we present KSRV, a framework for inferring spatial RNA velocity by integrating spatial transcriptomics with scRNA-seq data, enhancing data processing to better reconstruct cellular differentiation trajectories and enabling a spatially resolved depiction of cell-fate transitions. The core steps include: (1) independently perform nonlinear kernel PCA (Reverter et al., 2014) on scRNA-seq and spatial transcriptomics data to obtain their respective latent spaces, followed by alignment of these spaces; (2) infer the spliced and unspliced gene expression for each spatial spot by leveraging the gene expression profiles of neighboring single cells; (3) incorporate spatial location information to compute spatial RNA velocity vectors and reconstruct cell differentiation trajectories. KSRV enables the reconstruction of spatial differentiation trajectories at the single-cell resolution, and demonstrates the generalizability and biological interpretability across diverse datasets, offering a robust and versatile tool for studying spatial developmental dynamics.

## 2 Methods and materials

### 2.1 KSRV algorithm

**FIGURE 1:**
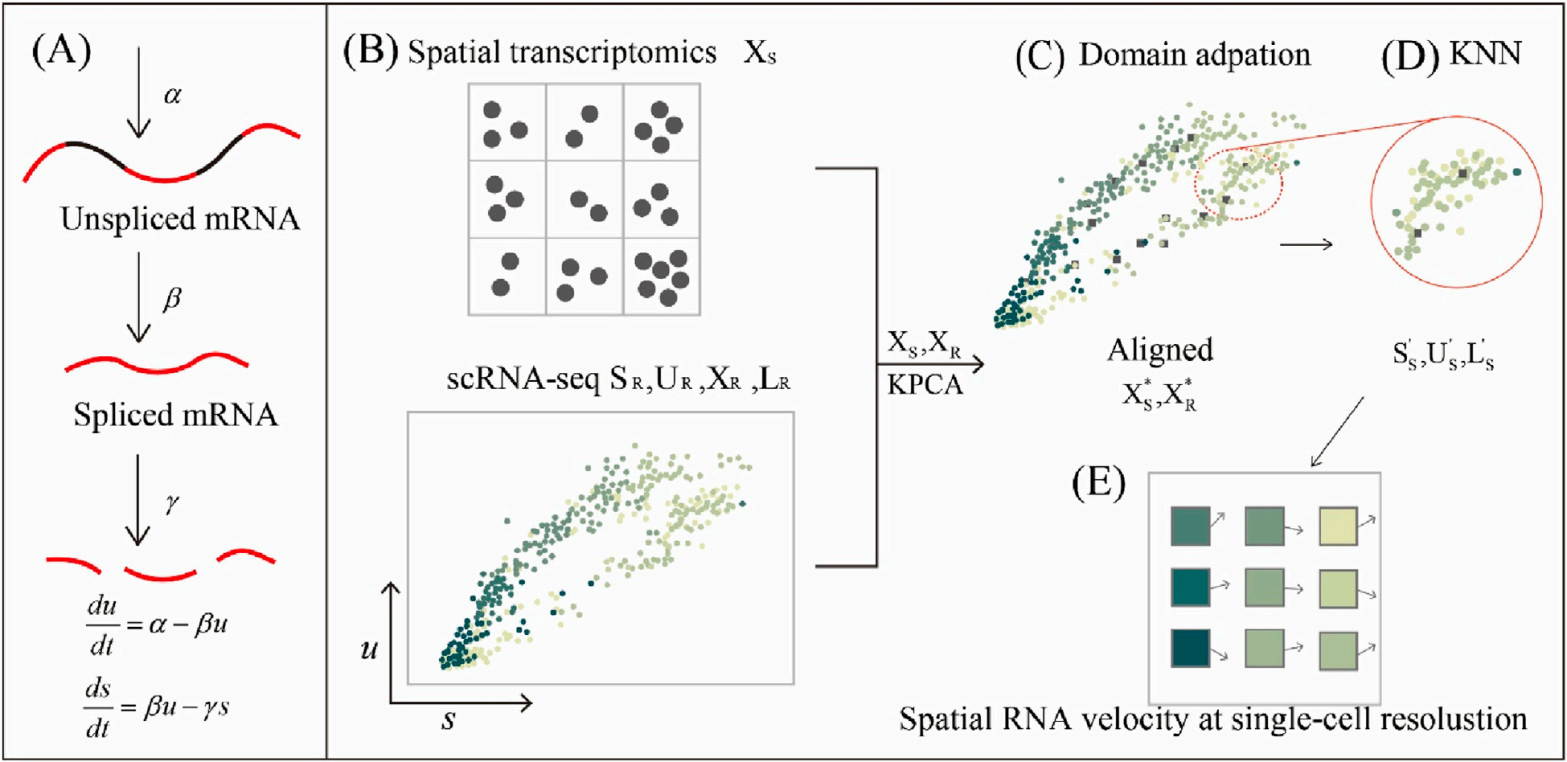
Overview of KSRV. **(A)** Transcriptional dynamics of genes. **(B)** Spatial transcriptomics data $X_{S}$ and reference scRNA-seq data $Q_{R}=\left\{\;S_{R},U_{R},X_{R},L_{R}\right\}$ as input. **(C)** Using domain adaptation with KPCA to integrate the two datasets $X_{S}$ and $X_{R}$ , generating aligned datasets $X_{S}^{*}$ and $X_{R}^{*}$ . **(D)** Using kNN regression based on the aligned datasets to predict spatial spliced $S_{S}^{\prime }$ and unspliced $U_{S}^{\prime }$ expression from scRNA-seq data, with labels $L_{S}^{\prime }$ . **(E)** Calculating RNA velocity vectors using the predicted $S_{S}^{\prime }$ and $U_{S}^{\prime }$ expressions, projecting them onto the tissue spatial coordinates to estimate spatial differentiation trajectories.

According to established models of transcriptional dynamics, genes are first transcribed into unspliced mRNA, which is then spliced into mature mRNA mRNA before being degraded (Luo et al., 2022). Based on this process, RNA velocity is defined as the first-order time derivative of spliced mRNA abundance (see Equation 1) (Aivazidis et al., 2025):

$$\left\{\begin{array}{c} \frac{du_{ij}}{dt_{i}}=\alpha_{ij}-\beta_{ij}u_{ij},\\ \frac{ds_{ij}}{t_{i}}=\beta_{ij}u_{ij}-\gamma_{ij}s_{ij}, \end{array}\right.$$ (1)

here, $\alpha_{ij}$ , $\beta_{ij}$ , and $\gamma_{ij}$ represent the transcription, splicing, and degradation dynamics rates of gene j in cell i , respectively. The variables $u_{ij}$ and $s_{ij}$ denote the abundance of unspliced and spliced mRNA, while $t_{i}$ represents pseudotime of cell i . However, applying this model to spatial transcriptomics (ST) data presents a major challenge, as most existing ST platforms do not distinguish between spliced and unspliced transcripts. To address this, we proposed a kernel-based framework for spatial RNA velocity inference (KSRV) that integrates scRNA-seq and ST data. As illustrated in Figure 1, the algorithm consists of three main steps:

Step 1 - scRNA-seq and ST data are independently projected into a nonlinear latent space via kernel PCA, and then aligned.
Step 2 - Based on aligned latent representations, KSRV predicts spliced and unspliced expression at each spatial spot by borrowing information from nearby single cells.
Step 3 - With the enriched data, spatial RNA velocity vectors are estimated and used to reconstruct cell differentiation trajectories in space at single-cell resolution.

#### 2.1.1 Integration

KSRV employs Kernel PCA to project single-cell data and ST data into a shared latent space (Briscik et al., 2023). First, the algorithm identifies the common gene set between the two datasets. To account for potential domain differences and mitigate batch effects, the PRECISE domain adaptation framework (Mourragui et al., 2019) is applied, aligning the distributions of single-cell and ST data prior to dimensionality reduction. Kernel PCA with a radial basis function (RBF) kernel, whose effectiveness for non-linear data has been validated, is then applied to each dataset separately, generating high-dimensional feature spaces and their corresponding kernel matrices (Wen et al., 2021). Following (He et al., 2025), the default value of the RBF kernel gamma was adopted in our framework, as it has been shown to perform robustly in similar applications. Subsequently, eigenvectors of these matrices are computed to extract the principal components. To align the datasets, singular value decomposition (SVD) is applied to orthogonalize the components, and only those with cosine similarity exceeding a threshold of 0.3 are retained. As shown in Supplementary Figure S2, this threshold was chosen based on sensitivity analysis, where a value >0.3 consistently yielded the best performance across datasets. Finally, both datasets are projected onto the resulting common latent space, achieving alignment while preserving non-linear gene expression patterns.

#### 2.1.2 Prediction of spatial transcriptome data

After alignment, the latent space is used to enrich the spatial transcriptomics data by inferring unmeasured spliced and unspliced gene expression. This is achieved via *k*-nearest neighbors (kNN) regression. Based on systematic evaluation across datasets (see Supplementary Figure S1), we set *k* = 50, which consistently maximized the similarity score and yielded robust predictions. For each spot i , its k nearest neighbors ( NN ) are identified from the aligned scRNA-seq data in the shared latent space, and the spliced $\left(S_{ig}^{\prime }\right)$ and unspliced $\left(U_{ig}^{\prime }\right)$ expression values of gene g in spot i are predicted as a weighted average across the neighbors cells j (Equations 2, 3):

$$S_{ig}^{\prime }=\sum_{j\in NN\left(i\right)}a_{ij}^{*}\times SR_{ig}$$ (2)

$$U_{ig}^{\prime }=\sum_{j\in NN\left(i\right)}a_{ij}^{*}\times {UR}_{ig}$$ (3)

here, $a_{ij}^{*}$ represents the weight between each spatial cell i and its j -th neighbor, which is inversely proportional to its cosine distance $d\left(i,j\right)$ to spot i ,

$$a_{ij}^{*}=\frac{a_{ij}}{k-1},\sum_{j\in NN\left(i\right)}a_{ij}^{*}=1$$ (4)

where $a_{ij}=1-\frac{d\left(i,j\right)}{\sum_{j\in NN\left(i\right)}d\left(i.j\right)},\sum_{j\in NN\left(i\right)}a_{ij}^{*}=1$ , and k denotes the number of nearest neighbors in Equation 4.

Similarly, KSRV also uses kNN regression to transfer cell type labels from scRNA-seq to spatial data. For spatial spot i and each cell type C , we sum the weights of k neighbors $\left(j\right.$ ) in scRNA-seq data labeled as C to compute a score $A_{iC}$ in Equation 5:

$$A_{iC}=\sum_{\begin{array}{c} j\in NN\left(i\right)\\ j\in C \end{array}}a_{ij}^{*}$$ (5)

where $\sum_{C}A_{iC}=1.$ The spatial spot i is then assigned the cell type with the highest score: $C_{i}=\arg\max_{C}A_{iC}$ .

#### 2.1.3 Evaluation metrics

Based on the predicted spliced and unspliced expression data, RNA velocity vectors for spatial cells are can be calculated. These vectors are then projected onto the tissue’s spatial coordinates to visualize cell dynamics in space.

To quantitatively evaluate the accuracy of the inferred differentiation trajectories, we calculate a weighted cosine similarity score between the estimated and reference RNA velocity vectors. The score is defined as:

$$Similarity\;Score=\sum_{i}\beta_{i}\times CS_{i}$$ (6)

where $\beta_{i}=\frac{M_{i}}{\sum_{j}M_{j}}$ is the normalized weight of vector magnitude, and $M_{j}$ is the magnitude of the velocity vector at position j , $CS_{i}$ in Equation 6 is the cosine similarity between the estimated and reference velocity vectors at position i .

### 2.2 Analysis of euclidean distance in cell space

We analyzed the variance of cells Euclidean distances from the origin over differentiation time in each dataset, and the positional differences measured by spatial transcriptomics are directly proportional to the Euclidean distances calculated in the two-dimensional plane. Differentiation time was divided into ten equal intervals. For each interval, we calculated the variance ( $\sigma^{2}$ ) of Euclidean distances of spot i (with the coordinates of $x_{i}$ and $y_{i}$ ) from the origin ( $d_{i}=\sqrt{x_{i}^{2}+y_{i}^{2}}$ ) as follows in Equation 7:

$$\sigma^{2}=\frac{1}{n}\sum_{i=1}^{n}{\left(d_{i}-\bar{d}\right)}^{2}$$ (7)

where n represent the number of spots in each time interval, and $\bar{d}$ represent the mean distance.

To capture how the variance of Euclidean distances changes over differentiation time, we fitted σ^2 at each time interval using a cubic polynomial curve:

$$\sigma^{2}=at^{3}+bt^{2}+ct+d,$$ (8)

where t is set as the median differentiation time within that interval.

Similarly, to examine the range of spot displacement over time, we divided the pseudo time into equal intervals and computed the 10th and 90th percentiles of Euclidean distances from the origin within each interval. These percentiles serve as robust proxies for the minimum and maximum distances, reducing the impact of outliers. We then fitted their temporal trends using the same cubic model described in Equation 8.

### 2.3 Analysis of regulatory factors of gene expression levels

To explore the contributions of temporal and spatial factors to gene expression, we introduced the concept of pseudo-spatiotemporal expression, which integrates both a cell developmental time and its spatial location.

The temporal component ( T ) is represented by latent time inferred via scVelo (Bergen et al., 2020), which better approximates real biological time than pseudotime. The spatial component ( D ) is captured using the Euclidean distance from each cell to a dataset-specific origin, thereby reducing spatial complexity to a one-dimensional value while preserving relative spatial information. Then, the average expression level $Y_{i}^{\prime }$ of spot i was calculated as a weighted combination of these two factors:

$$Y_{i}^{\prime }=\omega_{ji}T_{i}+\left(1-\omega_{ji}\right)D_{i}$$ (9)

Here, $T_{i}$ is the latent time, $D_{i}$ is the spatial distance, and $\omega_{i}\in \left[0,1\right]$ denotes the relative contribution of time versus space in Equation 9.

To estimate the values of ω , we assumed that all spots within the same cell type j share a common $\omega_{i}$ , based on the biological premise that cells of the same type tend to share similar regulatory dynamics (Yan et al., 2024). This assumption reduces model complexity and facilitates biological interpretation. The value of $\omega_{i}$ is then determined by minimizing the following loss function in Equation 10:

$$LOSS=\sum {\left(Y^{\prime }-Y\right)}^{2}$$ (10)

where Y represents the true mean non-zero expression across all genes at spot i . Users can obtain ω values directly using the built-in KSRV function, which implements this estimation procedure.

### 2.4 Description of the data set

We obtained a pair of datasets from the developing chicken heart, including 10x Visium spatial data and 10x Chromium scRNA-seq data from day 14 (Mantri et al., 2021). We ultimately obtained 1,967 spots and 12,295 genes for the Visium data, and 3,009 cells and 10,143 genes for the scRNA-seq data, along with the corresponding spliced and unspliced expressions. We obtained three spatial transcriptomics datasets (batches) measured from human osteosarcoma cells using MERFISH (Xia et al., 2019). The total Details, including total RNA counts, counts co-localized with the endoplasmic reticulum and nucleus, and spatial information, are provided in the Supplementary Material. Here, spliced and unspliced expressions are replaced by cytoplasmic and nuclear expressions, respectively. We used batch 1 (645 cells, 2,330 genes, and their spatial locations) as our spatial data, while batch 3 (323 cells, 12,903 genes) was used as simulated matched scRNA-seq data (ignoring the spatial locations of the cells).

For detailed information on Mouse Brain Development and Mouse Organogenesis (Lohoff et al., 2021; Pijuan-Sala et al., 2019), please refer to Table 1 and Supplementary Tables.

**TABLE 1:** Overview of the data sets used in this manuscript.

Dataset	Cell × gene (scRNA-seq)	Spot/Cell × gene (ST)	Technology (ST)	References
Developing chicken heart	3,009 × 10,143	1,967 × 12,295	Visium	Mantri, M. et al. (2021)
Human osteosarcoma	323 × 12,903	645 × 2,330	MERFISH	Xia, C. et al. (2019)
Developing mouse brain	40,733 × 16,907	4,628 × 119	HybISS	La Manno et al. (2021)
Mouse organogenesis	16,861 × 29,542	20,577 × 351	seqFISH	Pijuan-Sala, B. et al. (2019)

## 3 Results

### 3.1 Overview of KSRV

KSRV is a method for estimating RNA velocity at single-cell resolution in spatial transcriptomics by leveraging reference scRNA-seq data. The scRNA-seq dataset provides spliced ( $S_{R}$ ), unspliced ( $U_{R}$ ), and total ( $X_{R}$ ) gene expression, as well as optional metadata such as cell-type annotations $\left(L_{R}\right.$ ). To align the two modalities, we first apply PRECISE domain adaptation, which aligns the distributions of single-cell and spatial transcriptomics data and mitigates potential batch effects. This step ensures that the subsequent kernel PCA projection captures true biological similarity rather than technical variation. Kernel PCA is then applied to obtain a shared low-dimensional representation of both datasets. Using this aligned space, kNN regression is employed to transfer spliced and unspliced expression levels as well as cell-type labels from scRNA-seq to spatial spots. With the predicted spliced and unspliced expression, RNA velocity vectors were estimated for each spatial spot. These vectors are then projected onto tissue coordinates, revealing the spatial patterns of differentiation. Detailed implementation steps, including the full workflow and parameter settings, are provided in the Methods section. Additionally, the Supplementary Material provides a step-by-step illustration of KSRV applied to the chicken heart dataset as an example.

### 3.2 Evaluation of KSRV on two datasets

To assess the accuracy and robustness of KSRV, we conducted experiments on two datasets with ground-truth or reference RNA velocity: the 10x Visium dataset of developing chicken heart tissue and the MERFISH dataset of human osteosarcoma (U-2 OS) cells.

**FIGURE 2:**
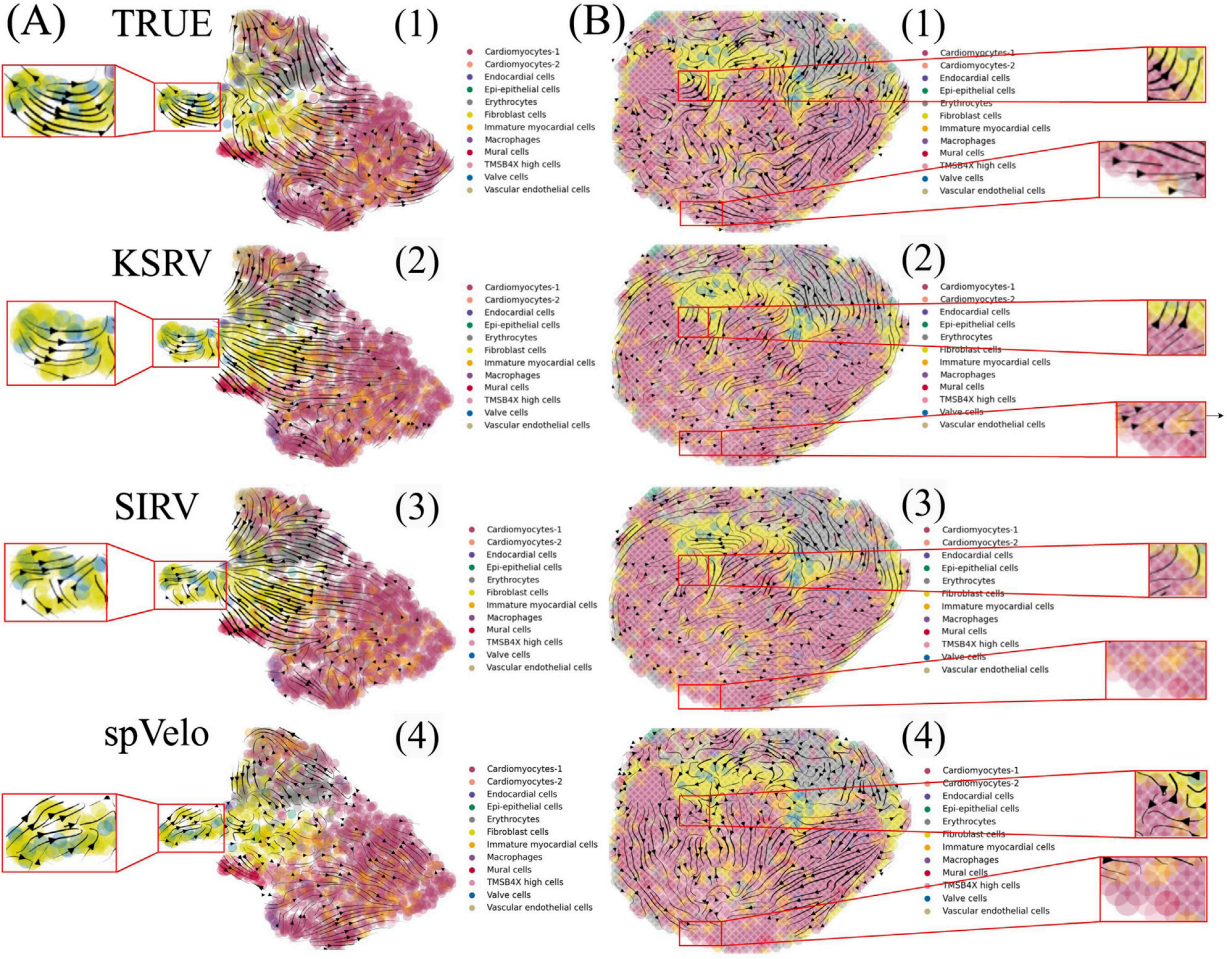
Comparison of RNA velocity inference across different methods. **(A)** Velocity projection in UMAP space. (1-4) RNA velocity estimated directly from ST data, inferred by KSRV, SIRV, and spVelo, respectively. **(B)** Corresponding velocity projections in spatial coordinates, shown in the same order as in **(A)**.

In the chicken heart dataset, each tissue spot contains both spliced and unspliced transcript reads, allowing for direct computation of reference RNA velocity using scVelo. These reference velocities were projected onto both UMAP space and spatial coordinates (Figures 2A,B), revealing clear directional trends of cellular differentiation. Notably, velocity projections in spatial coordinates more accurately reflected the biological organization of differentiation, due to preservation of the physical structure of the tissue. As shown in Figure 2A (4), KSRV also inferred RNA velocity for this dataset by integrating single-cell transcriptomic information into spatial domains, without relying on spliced and unspliced transcript reads from spatial data. The overall differentiation trajectory inferred by KSRV closely matched the reference velocity, demonstrating its ability to accurately capture the underlying dynamic patterns.

**FIGURE 3:**
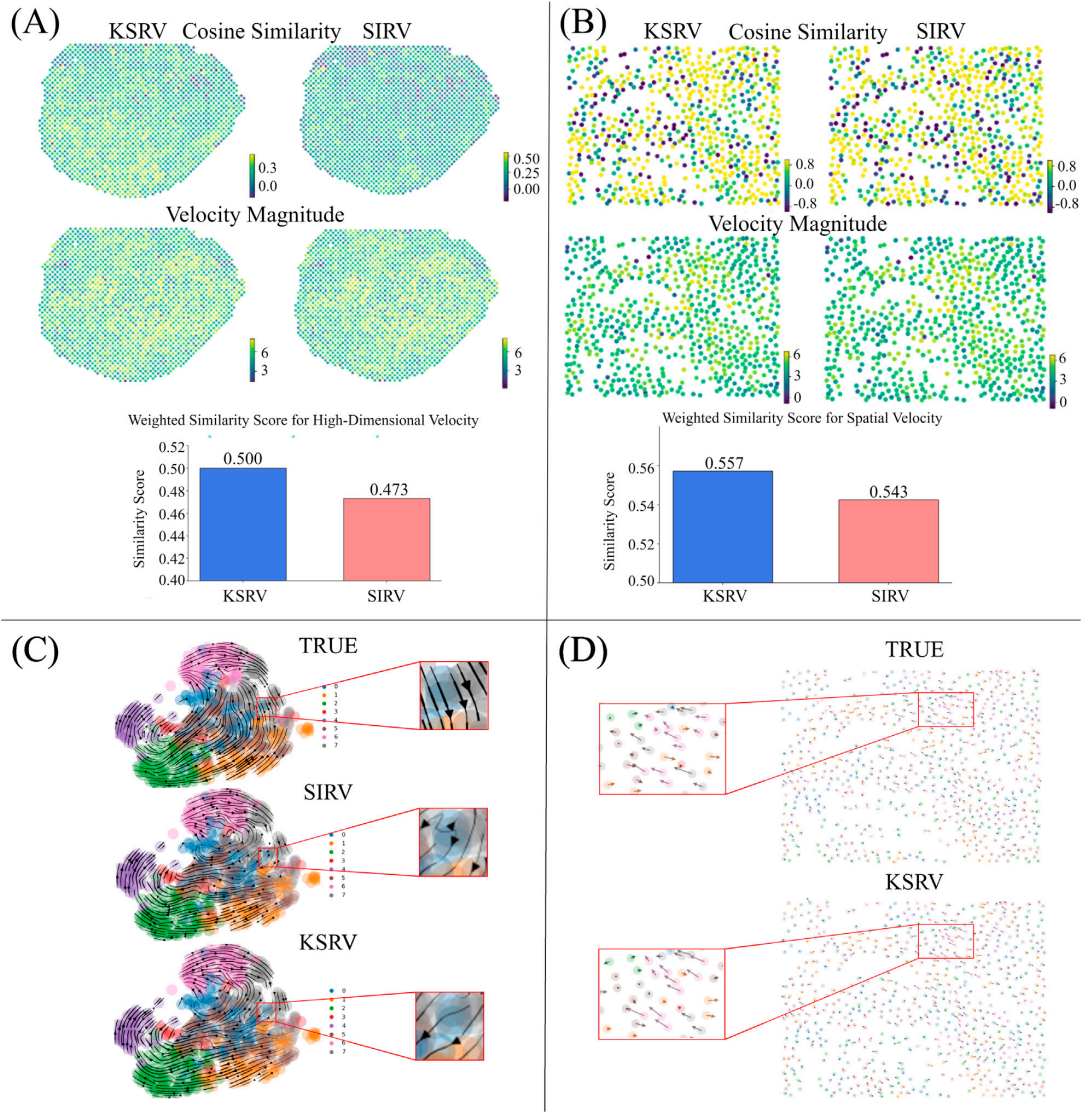
**(A)** The top, middle, and bottom panels respectively show the high-dimensional velocity similarity, two-dimensional velocity magnitude, and weighted similarity of high-dimensional velocity for the chicken heart dataset using the KSRV and SIRV methods. **(B)** The top panel shows the two-dimensional velocity similarity for U-2 OS using the two methods, while the remaining panels are the same as in **(A)**. **(C)** The top, middle and bottom are respectively the velocity flow of the real idle data of U-2 OS on UMAP, the velocity flow obtained by SIRV, and the velocity flow obtained by KSRV. **(D)** The upper and lower parts are respectively the velocity flow of the real idle data of U-2 OS in spatial coordinates and the velocity flow obtained by KSRV.

Similarly, we applied two existing methods, SIRV (Abdelaal et al., 2024), and spVelo (Long et al., 2025), to infer differentiation trajectories for this dataset (Figure 2A). While both methods produced trajectories that shared some similarity with the reference, notable discrepancies were observed in certain regions, particularly at the initial states. To quantitatively evaluate prediction accuracy, we computed cosine similarity and velocity magnitude between the predicted and reference velocities for each cell (Figure 3A). Across all cells, KSRV achieved significantly higher similarity scores (0.50) compared to both SIRV (0.47), highlighting its superior accuracy. In addition, Figures 2B, 3B illustrate the RNA velocity vectors and differentiation trajectories of cells at different spatial locations. Both KSRV and SIRV produced results that were broadly consistent with the reference trajectories. However, KSRV demonstrated superior accuracy in certain central and peripheral regions, leading to a higher similarity score (0.56) compared to SIRV (0.54). These results indicate that integrating single-cell transcriptomic data enables more precise inference of spatial RNA velocity at each spot, improving the fidelity of dynamic cellular state reconstruction.

To further evaluate the performance of KSRV, we applied it to a MERFISH dataset of the human osteosarcoma cell line U-2 OS. Although it does not distinguish between spliced and unspliced transcripts, cytoplasmic and nuclear mRNA signals can serve as proxies, assuming that spliced transcripts are enriched in the cytoplasm and unspliced transcripts in the nucleus. We first divided the MERFISH dataset into eight clusters and computed RNA velocity vectors based on cytoplasmic (spliced) and nuclear (unspliced) expression levels. To simulate matched single-cell RNA-seq data, we selected cells from other MERFISH batches while ignoring their spatial positions.

In this dataset, KPCA, a key component of KSRV, demonstrated clear advantages over traditional PCA in capturing velocity flow and differentiation trajectories (Figure 3C). As shown in Figure 3C, KPCA produced velocity vectors closely resemble the reference trajectories in both global patterns and local directional details (e.g., the red boxed region), while PCA (used by SIRV) showed notable deviations in several areas. Figure 3D further confirms that KPCA recapitulates the spatial structure of differentiation dynamics more accurately than PCA, with better alignment in both clustering patterns and directional flow.

To quantitatively assess accuracy, we computed the cosine similarity and Spearman correlation between predicted and observed gene expression levels, and averaged the values across all cells. The similarity scores of the KSRV method were 0.824 (cos) and 0.787 (Spearman) respectively, which were superior to those of the SIRV method. The latter had lower scores of 0.683 and 0.612 respectively under the same evaluation. These results support the accuracy and robustness of KSRV in reconstructing cellular differentiation dynamics from imaging-based spatial transcriptomics data.

### 3.3 Spatiotemporal dynamics of cell differentiation revealed by KSRV

**FIGURE 4:**
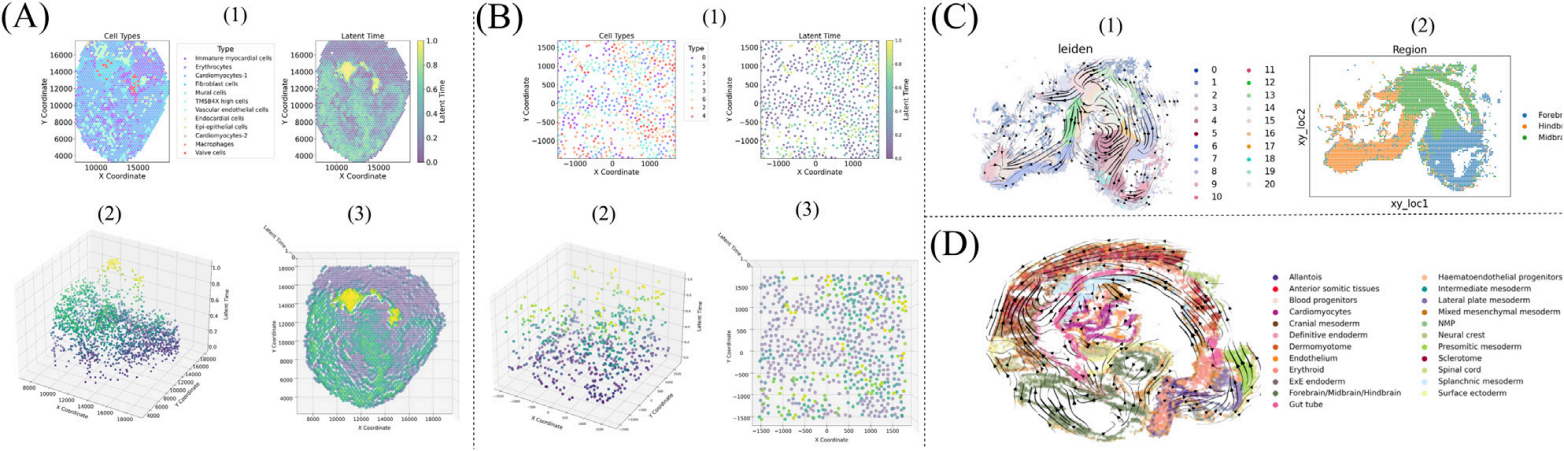
**(A)** Spatiotemporal differentiation relationships in the chicken heart. (1) Distribution map of cell types at different time points during cell differentiation. (2) and (3) are respectively the front view and top view of the spatial position distribution of cells over time. **(B)** Spatiotemporal differentiation relationships in U-2 OS. For detailed explanations, please refer to **(A)**. **(C)** Velocity flow and regional distribution during mouse brain development. **(D)** Velocity flow during mouse organogenesis.

KSRV permits joint visualization of cell type, differentiation time (pseudotime) and spatial location, offering an integrated view of tissue morphogenesis (Figure 4). In developing chicken heart tissue (Figure 4A), cell-type identities (panel 1) and pseudotime (panel 2) form overlapping spatial gradients: progenitor populations occupy the ventricular apex, whereas differentiated fibroblast and valve cells localise to atrioventricular and outflow regions. Re-mapping pseudotime onto the cell-type panel (panel 3) reproduces the same spatial pattern, confirming that cardiogenesis proceeds along a well-defined anatomical axis.

Similar spatial-pseudotemporal coherence is observed in the U-2 OS MERFISH data (Figure 4B), where eight transcriptional clusters arrange along a radial trajectory from the center outward, consistent with spatially organized transcript migration during osteo-sarcoma progression. Velocity vector fields inferred from two regions of embryonic mouse tissue further highlight KSRV’s ability to resolve fine-scale dynamic patterns (Figures 4C,D). In the developing brain (Figure 4C), inferred velocity flows converge near ventricular zones and diverge toward the cortical surface, aligning with established neurogenesis patterns (Stuart et al., 2019). These velocity fields not only visualize cell migration trajectories but also offer new perspectives on the spatial orchestration of differentiation and tissue formation.

To quantify the relative contributions of temporal versus spatial regulation, we modeled cell state progression as a linear combination of pseudotime and Euclidean distance (Table 2; Supplementary Table). In the chicken heart, early-stage differentiation is primarily time-driven, with immature myocardial and vascular endothelial cells showing high pseudotime weights (0.541 and 0.536). In contrast, late-stage fibroblast and valve cell lineages exhibit lower pseudotime weights (0.233 and 0.416), indicating stronger spatial dependence. In the U-2 OS dataset, early differentiation originates from cluster 0 with a pseudotime weight of 0.325, suggesting initial spatially constrained organization. To-ward the end of differentiation, cells accumulate in cluster 4, with a higher temporal weight of 0.614, indicating a shift toward pseudotime-dominated progression (Supplementary Table S1). These results demonstrate that KSRV effectively resolves both spatial and temporal components of differentiation dynamics across diverse tissues, providing a unified framework for dissecting developmental programs.

**TABLE 2:** ω in different cell types (Developing chicken heart).

Type	Omega
Cardiomyocytes-1	0.459
Cardiomyocytes-2	0.5
Endocardial cells	0.048
Epi-epithelial cells	0.106
Erythrocytes	0.317
Fibroblast cells	0.233
Immature myocardial cells	0.541
Macrophages	0.783
Mural cells	0.278
TMSB4X high cells	0.604
Valve cells	0.416
Vascular endothelial cells	0.536

### 3.4 Temporal dynamics of euclidean distance during cell differentiation

**FIGURE 5:**
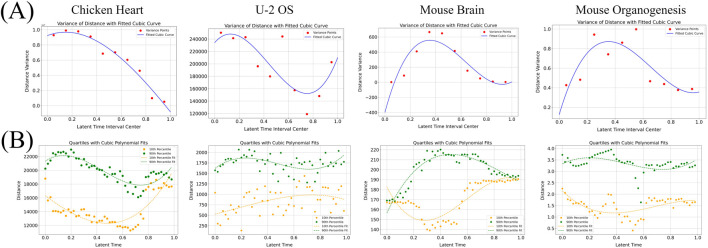
**(A)** Variance of Euclidean distance. **(B)** Extremum of Euclidean distance. The relationship between variance and extremum is closely related to the development of time. Take the chicken heart dataset as an example. As time goes by, the variance between distances decreases, indicating that cell differentiation tends to be concentrated, that is, the extreme values are getting closer and closer.

To further dissect the spatial organization of differentiation, we analyzed changes in Euclidean distance from the origin over pseudotime across four datasets: chicken heart, U-2 OS, mouse brain, and mouse organogenesis (Figure 5). In the chicken heart dataset, Euclidean distance variance decreases steadily with pseudotime (Figure 5A, top left), suggesting that cells gradually con-verge spatially during differentiation. This spatial consolidation aligns with the patterns observed in Figure 4A, where terminal fibro-blast and valve cells occupy anatomically restricted regions. Figure 5B further supports this observation: early in differentiation, cells exhibit a broad range of distances from the origin, indicating spatial dispersion; later, the spread narrows, consistent with terminal spatial convergence.

Conversely, in the U-2 OS dataset, distance variance increases with pseudotime (Figure 5A, top right), indicating progressive spatial dispersion. As seen in Figure 4B, terminal cell states are spatially scattered, reflecting a less constrained spatial organization during late-stage osteosarcoma progression. Figure 5B shows sustained variability in Euclidean distances throughout the trajectory, con-firming that cells remain spatially distributed across the differentiation continuum.

These results highlight contrasting spatial differentiation dynamics across tissues. While chicken heart development exhibits increasing spatial organization and compartmentalization, U-2 OS cells maintain spatial heterogeneity, possibly reflecting differences in tissue architecture or pathological state.

## 4 Discussions and conclusion

In this paper, a new method KSRV is proposed to infer RNA velocity in a spatial context at single-cell resolution. This method can combine single-cell data with spatial transcriptomics data. By leveraging domain adaptation and Kernel PCA, it maps integrate the information from single-cell sequencing data onto spatial transcriptomics data. Therefore, the spliced and unspliced data can be obtained in gene expression levels. And also it can obtain the cell type at the point/cell level. By anchoring these vectors to physical coordinates, KSRV reveals the spatiotemporal flow of differentiation within intact tissue. Unlike SIRV, we employ Kernel PCA to better handle non-linear data and thereby construct a more accurate velocity flow.

Benchmarking on 10x Visium chicken-heart and MERFISH U-2 OS datasets shows that KSRV reproduces reference velocity fields with substantially higher similarity score than the current SIRV method. This validates the superiority of Kernel PCA in capturing velocity flow dynamics and differentiation characteristics. Beyond validation, KSRV mapped coherent lineage streams in developing mouse brain and organogenesis sections and quantified how spatial convergence (chicken heart) or dispersion (U-2 OS) unfolds over pseudotime via Euclidean-distance analysis. These results demonstrate that KSRV not only improves velocity prediction accuracy but also delivers mechanistic insight into how temporal and spatial cues jointly shape cell-state transitions, information essential for dissecting developmental programmes and disease progression.

Despite the significant progress made by KSRV, there are some limitations that need to be specifically pointed out. Notably, when projecting high-dimensional RNA velocity vectors onto a two-dimensional coordinate system, cells may be forced to point towards neighboring cells, potentially leading to the emergence of artifacts. In the current implementation, KSRV employs a traditional fusion strategy, KPCA, to integrate spatial transcriptomics (ST) and scRNA-seq data. While KPCA is effective for aligning the two datasets based on gene expression, it does not explicitly leverage spatial relationships within ST data, potentially limiting its ability to capture spatially structured biological variation. Moreover, KSRV does not perform feature selection prior to data integration, in order to retain as many shared genes as possible and ensure sufficient information for alignment and RNA velocity inference. Nevertheless, systematic feature selection, either unimodal methods such as GeneClust (Deng et al., 2023) for scRNA-seq or SpatialDE (Svensson et al., 2018) for spatial data, or multimodal approaches such as LEGEND (Deng et al., 2024), could help reduce noise, improve computational efficiency, and highlight biologically informative genes. Although the current KPCA-based fusion strategy demonstrates satisfactory performance, future work could explore more advanced alignment methods that explicitly incorporate spatial structure, such as STANDS (Xu et al., 2024), DSTG (Song and Su, 2021), or general-purpose integration tools like Harmony (Korsunsky et al., 2019). Such improvements, combined with feature selection strategies, could further enhance KSRV’s robustness, accuracy, and biological interpretability across diverse datasets and conditions.

## Data Availability

The original contributions presented in the study are included in the article/Supplementary Material, further inquiries can be directed to the corresponding authors. All code about KSRV can be downloaded from https://github.com/YanYan116/KSRV.
