# Supplementary material for: KSRV: a Kernel PCA-Based framework for inferring spatial RNA velocity at single-cell resolution
Source: Front Genet. 2025 Nov 7;16:1695803. doi: 10.3389/fgene.2025.1695803 (PMC12634034; doi:10.3389/fgene.2025.1695803)
Supplement: Supplementary file 1 [file Supplementaryfile1.docx]

*Supplementary Material*

KSRV: A Kernel PCA-Based Framework for Inferring Spatial RNA Velocity at Single-Cell Resolution

# Supplementary Data

## Developing chicken heart

We obtained a pair of datasets from the developing chicken heart, including 10x Visium spatial data and 10x Chromium scRNA-seq data from day 14 (Mantri, M. et al. 2021). The count matrices provided by the authors did not include spliced and unspliced expressions. Therefore, we downloaded the fastq files from GEO (GSE149457) and re-aligned the reads to the reference genome (GRCg6a) using kallisto/BUStools, distinguishing between intronic and exonic reads. Using the same cell/spot barcode list provided in the count matrices by the authors, we ultimately obtained 1,967 spots and 12,295 genes for the Visium data, and 3,009 cells and 10,143 genes for the scRNA-seq data, along with the corresponding spliced and unspliced expressions (Table 1).

## Human osteosarcoma (U-2 OS)

We obtained three spatial transcriptomics datasets (batches) measured from human osteosarcoma cells using MERFISH (Abdelaal, T. et al. 2024; Xia, C. et al. 2019). The total RNA counts for each gene in each cell, as well as the counts co-localized with the endoplasmic reticulum and nucleus, are provided in datasets S12, S13, and S14, respectively, while the spatial information for each cell is provided in dataset S15. Here, spliced and unspliced expressions are replaced by cytoplasmic and nuclear expressions, respectively. We used batch 1 (645 cells, 2,330 genes, and their spatial locations) as our spatial data, while batch 3 (323 cells, 12,903 genes) was used as simulated matched scRNA-seq data (ignoring the spatial locations of the cells).

## Developing mouse brain

We utilized spatial transcriptomics and scRNA-seq datasets from the developing mouse brain atlas. The datasets were downloaded. The spatial transcriptomics data measured the expression of 119 genes in 4,628 cells in an E10.5 mouse embryo using the HybISS protocol (La Manno, G. et al. 2021; Abdelaal, T. et al. 2024). Among the 25 different spatial slices provided by the authors, we selected the "40 μm" slice, which contains clear brain structures. Cell segmentation was not provided in the data, but we used the voxelized version of the data (provided by the authors), which summarizes spatial gene expression on a 30,000-pixel 2D grid. The scRNA-seq data depicted gene expression in developing mouse brain tissue from E7 to E18. To match the HybISS data, we used only data from E10 and E11. After processing, we obtained 40,733 cells and 16,907 genes. Additionally, the scRNA-seq data included multiple metadata labels, and we focused on the labels indicating regions (forebrain, midbrain, and hindbrain) and cell identities (the "Subclass" annotation).

## Mouse organogenesis

We used three spatial datasets measured using the seqFISH protocol, representing three slices from the same mouse embryo (Lohoff, T. et al. 2022; Abdelaal, T. et al. 2024). These three datasets contain a total of 52,568 cells (19,451, 14,891, and 23,194 cells in Embryo1, Embryo2, and Embryo3, respectively) and analyze the expression of 351 genes. We selected the Gastrulation Atlas (Pijuan-Sala, B. et al. 2019) as the reference scRNA-seq data for mouse organogenesis. We downloaded the complete atlas data from the Mouse Gastrulation Data R package, excluded cells without cell type annotations, and selected only E8.5 to match the seqFISH spatial data. In total, we analyzed 16,861 cells and 29,452 genes.

# Detailed Workflow of KSRV on the Chicken Heart Dataset

As shown in Supplementary Fig. 3, we illustrate the step-by-step application of KSRV to the chicken heart dataset in eight steps.

**Step 1 - Gene selection**. Identify the common genes between the spatial transcriptomics (ST) data and single-cell RNA-seq (scRNA-seq) data. The ST data has dimensions 1967×12295, and the scRNA-seq data has dimensions 3009×10143. A total of 9836 genes are shared, resulting in matrices A (1967×9836) and B (3009×9836) for ST and scRNA-seq, respectively.

**Step 2 - Domain adaptation**. To account for potential domain differences and mitigate batch effects between the ST and scRNA-seq datasets, the PRECISE domain adaptation framework (Mourragui et al., 2019) is applied. This aligns the distributions of the two datasets, ensuring that subsequent dimensionality reduction captures true biological variation rather than technical differences.

**Step 3 - Dimensionality reduction.** Apply kernel PCA (KPCA) to both datasets using the selected common genes, retaining 50 principal components. After KPCA, the ST matrix becomes 9836×50(denoted as ST), and the single-cell matrix 9836×50(denoted as SC).

**Step 4 - Similarity computation.** Normalize the ST and SC matrices, and compute the cosine similarity matrix *C* (50×50) between their principal components to quantify the similarity between the two datasets.

**Step 5 - Selection of effective components.** Perform singular value decomposition (SVD) on C, and retain singular values greater than 0.3, resulting in 10 selected components ${(b}_{1},b_{2},\cdots,b_{10})$. The corresponding dimensionality-reduced information from the scRNA-seq data is preserved as common information *S* (9836×10).

**Step 6 - Alignment in latent space.** Map both datasets through the selected common components into the shared latent space, achieving alignment while preserving key biological variation.

**Step 7 - kNN-based expression transfer.** In the latent space, for each spatial spot, identify its 50 nearest neighbors in the scRNA-seq data. Predict spliced and unspliced gene expression by weighted averaging across neighbors:

$S_{ig}^{'}=\sum_{j\in NN(i)} a_{ij}^{*}\times SR_{ig},$ (1)

$U_{ig}^{'}=\sum_{j\in NN(i)} a_{ij}^{*}\times{UR}_{ig},$ (2)

where, the weight $a_{ij}^{*}$ is inversely proportional to the cosine distance $d(i,j)$ between spot $i$ and neighbors,

$a_{ij}^{*}=\frac{a_{ij}}{k-1}, \sum_{j\in NN(i)} a_{ij}^{*}=1.$ (3)

where $a_{ij}=1-\frac{d\left( i,j \right)}{\sum_{j\in NN\left( i \right)} d\left( i.j \right)}, \sum_{j\in NN\left( i \right)} a_{ij}^{*}=1$ , and $k$ denotes the number of nearest neighbors.

**Step 8 - RNA velocity estimation and visualization.** Using the predicted spliced and unspliced expression values, compute RNA velocity vectors for each spatial spot, and project them onto tissue coordinates to reveal spatial patterns of differentiation.

# Supplementary Figures and Tables

## Supplementary Figures


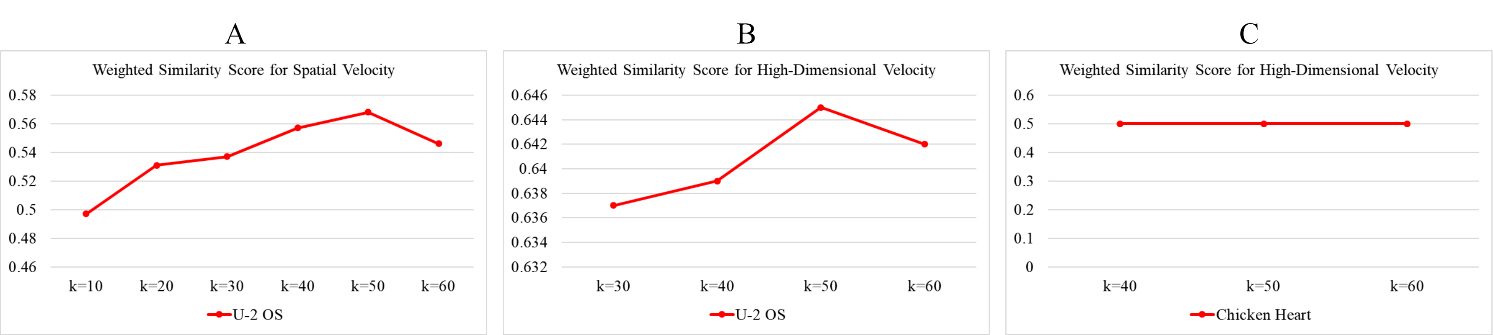


**Supplementary Figure 1.** **Analysis of the effect of different *k* values.** (A) Weighted similarity score for spatial velocity in the U-2 OS dataset. (B) Weighted similarity score for high-dimensional velocity in the U-2 OS dataset. (C) Weighted similarity score for high-dimensional velocity in the Chicken Heart dataset. In both datasets, the similarity score reached its maximum when k=50, indicating that this value provides the most robust and consistent performance. Therefore, k=50 was adopted in our framework.


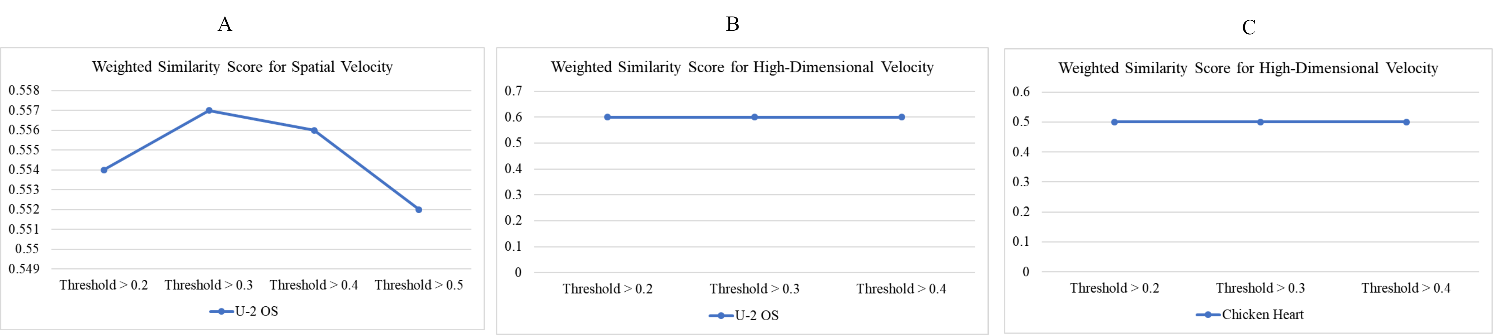


**Supplementary Figure 2.** **Analysis of the effect of different threshold values.** (A) Weighted similarity score for spatial velocity in the U-2 OS dataset, which reached its maximum when the threshold exceeded 0.3. (B) Weighted similarity score for high-dimensional velocity in the U-2 OS dataset and (C) in the Chicken Heart dataset, both showing minimal changes across different threshold values. These results indicate that a threshold > 0.3 provides robust performance and was therefore adopted in our framework.


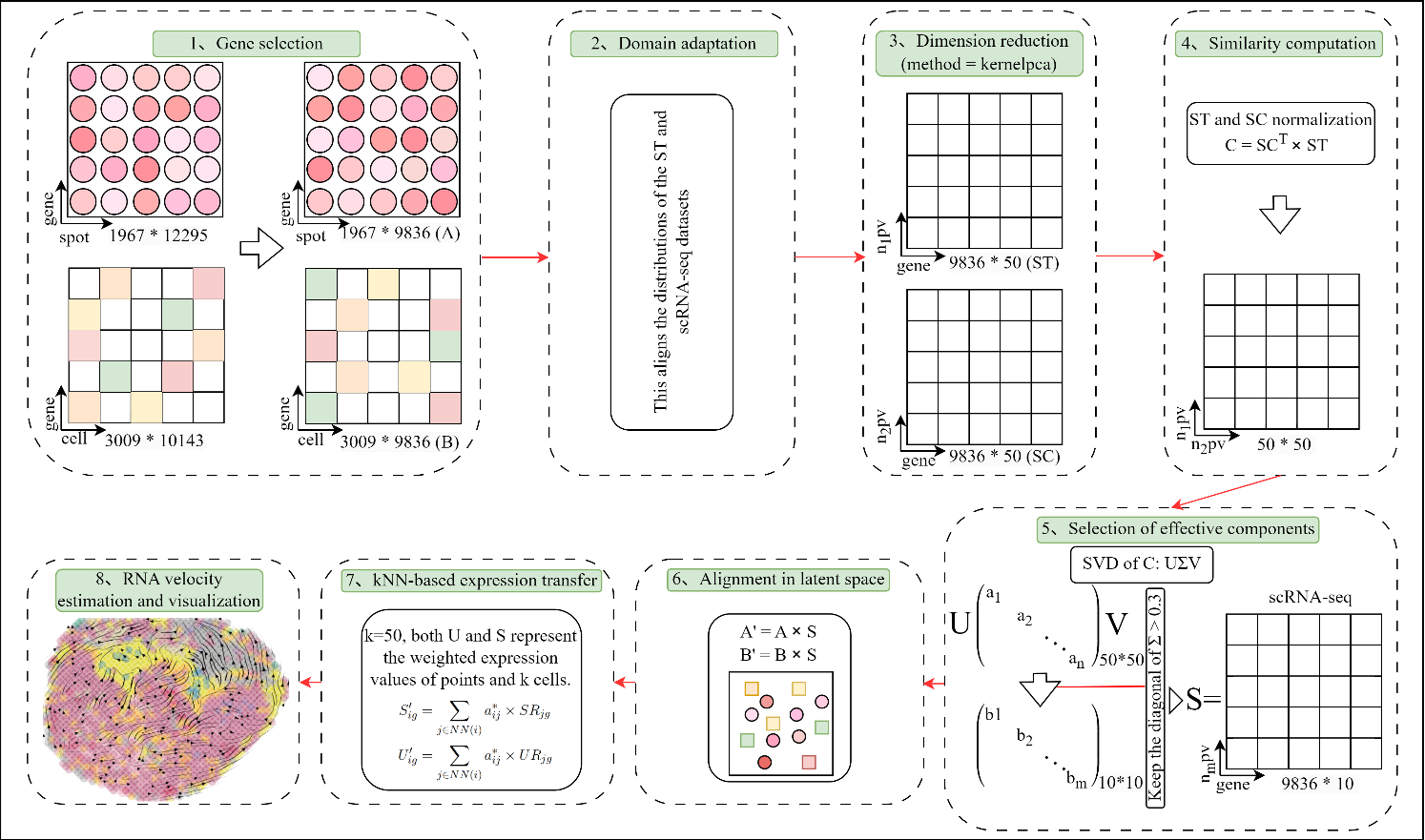


**Supplementary Figure 3**. The step-by-step application of KSRV to the chicken heart dataset.

## Supplementary Tables

**Supplementary Table 1.** $\omega$ in different cell types (U-2 OS)

| Type | omega |
| --- | --- |
| 0 | 0.325 |
| 1 | 0.405 |
| 2 | 0.457 |
| 3 | 0.392 |
| 4 | 0.614 |
| 5 | 0.388 |
| 6 | 0.488 |
| 7 | 0.486 |

**Supplementary Table 2.** $\omega$ in different cell types (Mouse organogenesis)

| Type | omega | Type | omega |
| --- | --- | --- | --- |
| Allantois | 0.251 | Haematoendothelial progenitors | 0.367 |
| Anterior somatic tissues | 0.267 | Intermediate mesoderm | 0.167 |
| Blood progenitors | 0.256 | Lateral plate mesoderm | 0 |
| Cardiomyocytes | 0.294 | Mixed mesenchymal mesoderm | 0.277 |
| Cranial mesoderm | 0.250 | NMP | 0.134 |
| Definitive endoderm | 0.348 | Neural crest | 0.289 |
| Dermomyotome | 0.214 | Presomitic mesoderm | 0.108 |
| Endothelium | 0.170 | Sclerotome | 0.342 |
| Erythroid | 0.245 | Spinal cord | 0.173 |
| ExE endoderm | 0.302 | Splanchnic mesoderm | 0.256 |
| Forebrain/Midbrain/Hindbrain | 0.075 | Surface ectoderm | 0.274 |
| Gut tube | 0.131 |  |  |

**Supplementary Table 3.** $\boldsymbol{\omega}$ **in different cell types (Developing mouse brain)**

| Type | omega | Type | omega |
| --- | --- | --- | --- |
| Angioblast | 0.593 | Hypothalamic floor-plate like | 0.612 |
| Anteromedial cerebral pole | 0.362 | Hypothalamus | 0.857 |
| Cajal-Retzius | 0 | Lateral nasal pit | 0.5 |
| Choroid plexus | 0.190 | Mesenchyme | 0.367 |
| Cortical hem | 0.246 | Midbrain | 0.136 |
| Dorsal diencephalon | 0.133 | Midbrain GABAergic | 0.262 |
| Dorsal forebrain | 0.133 | Midbrain basal plate | 0.308 |
| Dorsal hindbrain | 0 | Midbrain floor plate | 0.385 |
| Dorsal midbrain | 0.389 | Midbrain glutamatergic | 0.065 |
| Early choroid plexus | 0.3 | Midbrain-hindbrain boundary | 0.301 |
| Early fibroblasts | 0.347 | Mixed region | 0.191 |
| Early macrophage | 0.176 | Motor neuron | 0.460 |
| Erythroid progenitor | 0.214 | Neural crest | 0.361 |
| Forebrain | 0.213 | Neuronal intermediate progenitor | 0 |
| Forebrain GABAergic | 0.529 | Olfactory pit | 0 |
| Forebrain glutamatergic | 0.1759 | Optic cup | 0.757 |
| Fourth-ventricle roof plate | 0.240 | Roof plate | 0.251 |
| Hindbrain | 0.018 | Sensory neuron | 0.047 |
| Hindbrain GABAergic | 0.459 | Surface ectoderm | 0.408 |
| Hindbrain floor plate | 0.285 | Undefined | 0.540 |
| Hindbrain glutamatergic | 0 | Ventral hindbrain | 0.188 |
| Hindbrain glycinergic | 0.189 | Ventral midbrain | 0.006 |
| Hindbrain roof plate | 0.023 | Zona limitans intrathalamica | 1 |

# REFERENCES

Abdelaal, T., Grossouw, L. M., Pasterkamp, R. J., Lelieveldt, B. P. F., Reinders, M. J. T., and Mahfouz, A. (2024). SIRV: spatial inference of RNA velocity at the single-cell resolution. NAR Genomics and Bioinformatics 6. doi:10.1093/nargab/lqae100

La Manno, G., Siletti, K., Furlan, A., Gyllborg, D., Vinsland, E., Mossi Albiach, A., et al. (2021). Molecular architecture of the developing mouse brain. Nature 596, 92–96. doi:10.1038/s41586-021-03775-x

Lohoff, T., Ghazanfar, S., Missarova, A., Koulena, N., Pierson, N., Griffiths, J. A., et al. (2021). Integration of spatial and single-cell transcriptomic data elucidates mouse organogenesis. Nature Biotechnology 40, 74-85. doi:10.1038/s41587-021-01006-2

Mantri, M., Scuderi, G. J., Abedini-Nassab, R., Wang, M. F. Z., McKellar, D., Shi, H., et al. (2021). Spatiotemporal single-cell RNA sequencing of developing chicken hearts identifies interplay between cellular differentiation and morphogenesis. Nature Communications 12. doi:10.1038/ s41467-021-21892-z

Xia, C., Fan, J., Emanuel, G., Hao, J., and Zhuang, X. (2019). Spatial transcriptome profiling by MERFISH reveals subcellular RNA compartmentalization and cell cycle-dependent gene expression. Proceedings of the National Academy of Sciences 116, 19490-19499. doi:10.1073/pnas.1912459116
